# Effect of a genetically engineered interferon-alpha versus traditional interferon-alpha in the treatment of moderate-to-severe COVID-19: a randomised clinical trial

**DOI:** 10.1080/07853890.2021.1890329

**Published:** 2021-02-23

**Authors:** Chuan Li, Fengming Luo, Chengwu Liu, Nian Xiong, Zhihua Xu, Wei Zhang, Ming Yang, Ye Wang, Dan Liu, Chao Yu, Jia Zeng, Li Zhang, Duo Li, Yanbin Liu, Mei Feng, Ruoyang Liu, Jiandong Mei, Senyi Deng, Zhen Zeng, Yuanhong He, Haiyan Liu, Zhengyu Shi, Meng Duan, Deying Kang, Jiayu Liao, Weimin Li, Lunxu Liu

**Affiliations:** a Department of Thoracic Surgery, West China Hospital, Sichuan University, Chengdu, China; b Department of Pulmonary and Critical Care Medicine, West China Hospital, Sichuan University, Chengdu, China; c Department of Neurology, Union Hospital, Tongji Medical College, Huazhong University of Science and Technology, Wuhan, China; d Wuhan Red Cross Hospital, Wuhan, China; e Department of Critical Care Medicine, Mianyang Central Hospital, Mianyang, China; f Department of Respiratory and Critical Care Medicine, First Affiliated Hospital, The Second Military Medical University, Shanghai, China; g Department of Infectious Diseases, Guanggu District, the Maternal and Child Health Hospital of Hubei Province, Wuhan, China; h Department of Respiratory Medicine, The Public Health Clinical Center of Chengdu, Chengdu, China; i Department of Respiratory and Critical Care Medicine, Naval Hospital of Eastern Theater of PLA, Zhoushan, China; j Department of Aviation Disease, Naval medical center of PLA, the Second Military Medical University, Shanghai, China; k Department of Respiratory Disease, Wuhan Red Cross Hospital, Wuhan, China; l Department of Respiratory Disease, The Affiliated Hospital of Southwest Medical University, Luzhou, China; m Center of Infectious Diseases, West China Hospital, Sichuan University, Chengdu, China; n Department of Respiratory Disease, Sichuan Second Hospital of T. C. M, Chengdu, China; o Department of Infectious Disease, The Public Health Clinical Center of Chengdu, Chengdu, China; p Department of Tuberculosis, The Public Health Clinical Center of Chengdu, Chengdu, China; q Department of Liver Disease, The Public Health Clinical Center of Chengdu, Chengdu, China; r Department of Evidence based Medicine and Clinical Epidemiology, West China Hospital, Sichuan University, Chengdu, China; s Department of Bioengineering, Bourns College of Engineering, University of California, Riverside, CA, USA; t The West China-California Research Center for Predictive Intervention Medicine, West China hospital, Sichuan University, Chengdu, China

**Keywords:** COVID-19, SARS-CoV-2, interferon-alpha, recombinant super-compound interferon, treatment

## Abstract

**Background:**

There are few effective therapies for coronavirus disease 2019 (COVID-19) upon the outbreak of the pandemic. To compare the effectiveness of a novel genetically engineered recombinant super-compound interferon (rSIFN-co) with traditional interferon-alpha added to baseline antiviral agents (lopinavir–ritonavir or umifenovir) for the treatment of moderate-to-severe COVID-19.

**Method:**

In this multicenter randomized (1:1) trial, patients hospitalized with moderate-to-severe COVID-19 received either rSIFN-co nebulization or interferon-alpha nebulization added to baseline antiviral agents for no more than 28 days. The primary endpoint was the time to clinical improvement. Secondary endpoints included the overall rate of clinical improvement assessed on day 28, the time to radiological improvement and virus nucleic acid negative conversion.

**Results:**

A total of 94 patients were included in the safety set (46 patients assigned to rSIFN-co group, 48 to interferon-alpha group). The time to clinical improvement was 11.5 days versus 14.0 days (95% CI 1.10 to 2.81, *p* = .019); the overall rate of clinical improvement on day 28 was 93.5% versus 77.1% (difference, 16.4%; 95% CI 3% to 30%); the time to radiological improvement was 8.0 days versus 10.0 days (*p* = .002), the time to virus nucleic acid negative conversion was 7.0 days versus 10.0 days (*p* = .018) in the rSIFN-co and interferon alpha arms, respectively. Adverse events were balanced with no deaths among groups.

**Conclusions and relevance:**

rSIFN-co was associated with a shorter time of clinical improvement than traditional interferon-alpha in the treatment of moderate-to-severe COVID-19 when combined with baseline antiviral agents. rSIFN-co therapy alone or combined with other antiviral therapy is worth to be further studied.

**Key messages**
There are few effective therapies for coronavirus disease 2019 (COVID-19) upon the outbreak of the pandemic. Interferon alphas, by inducing both innate and adaptive immune responses, have shown clinical efficacy in treating severe acute respiratory syndrome coronavirus and Middle East respiratory syndrome coronavirus.
In this multicenter, head-to-head, randomized, clinical trial which included 94 participants with moderate-to-severe COVID-19, the rSIFN-co plus antiviral agents (lopinavir–ritonavir or umifenovir) was associated with a shorter time of clinical improvement than interferon-alpha plus antiviral agents.

## Introduction

The ongoing coronavirus disease 2019 (COVID-19) pandemic caused by the severe acute respiratory syndrome coronavirus 2 (SARS-CoV-2) has affected more than fifty-two million people worldwide as of November 13, 2020 [1]. Although most of the infections have been self-limited, about 20% of the infected adults have been found to develop severe pneumonia or a critical illness which, in some cases, has led to death [2–4]. Thus far, treatment strategies have included standard supportive care, corticosteroids, intravenous immunoglobulin, and empirical or repurposed antiviral therapies (e.g. remdesivir, ribavirin, lopinavir–ritonavir, umifenovir, and interferons, etc.) [5–10].

Interferon-alphas, by inducing both innate and adaptive immune responses, have shown clinical efficacy in treating various viral infections, such as SARS-Cov and Middle East respiratory syndrome coronavirus (MERS-Cov) [11,12]. During the time period of this study, there were no definitely effective antiviral therapies in treating patients with COVID-19. Interferon-alpha nebulization is empirically recommended for the treatment of SARS-CoV-2 pneumonia by the *Diagnosis and Treatment Protocol for Novel Coronavirus Pneumonia* released by the National Health Commission of China [13].

Recombinant super-compound interferon (rSIFN-co) is a new genetically engineered type I interferon that was created by changing 65 bases of 60 amino acid genetic codes of interferon-alphacon-1 without changing its amino acid composition. The changes altered the protein’s spatial conformation, which led to 20 times stronger antiviral activity (including against SARS-CoV), and reduced toxicity and side effects as compared with its prototype [14–16]. rSIFN-co can be safely used in large doses (each dose can be >10 million international units [IU]), making it possible to treat some viral diseases or tumours that require large doses of interferon [14–18]. Therefore, rSIFN-co was considered as a possible therapeutic option for the treatment of COVID-19 [18].

We conducted a multicenter, head-to-head, randomized clinical trial to compare the effect of rSIFN-co with traditional interferon-alpha in hospital-admitted adult patients presenting with moderate-to-severe COVID-19.

## Methods

### Study design and participants

This was a multicenter, head-to-head, randomized, single-blind, clinical trial conducted from February 10, 2020, to April 5, 2020. We recruited patients from five hospitals in Wuhan city, Hubei province, and in Chengdu city, Sichuan province, China.

Eligible patients were males and non-pregnant females aged 18 years or older, diagnosed with moderate-to-severe COVID-19 pneumonia according to the *Diagnosis and Treatment Protocol for Novel Coronavirus Pneumonia* released by the National Health Commission of China (Supplementary Table 1) [13]. Moderate COVID-19 patients were featured by fever, respiratory symptoms, and radiographic pneumonia, while severe COVID-19 patients featured by any of the following signs: dyspnoea, respiratory frequency ≥30/minute, oxygen saturation ≤94%, and PaO2/FiO2 ratio <300mmHg. The diagnosis of COVID-19 pneumonia was confirmed with reverse-transcription polymerase chain reaction (RT-PCR) testing of SARS-CoV-2 nucleic acid by nasopharyngeal swab test and chest computed tomography (CT) scans. Patients who received symptomatic treatment and/or supportive care before enrolment but had no clinical improvement were also involved. We excluded patients if they presented with any condition that would not allow the protocol to be followed safely; had a history of allergy or hypersensitivity to interferons or any of the ingredients used in this trial; had a history of myocardial infarction and other serious cardiovascular diseases; were unable to receive nebulized compound; and/or voluntarily requested to withdraw from the trial.

The patients who m*et al*l the following criteria were considered cured and could be discharged from the hospital, if their body temperature remained normal for at least three days, their respiratory symptoms relieved, and they obtained two consecutive negative tests for SARS-CoV-2 (interval between tests was more than 24 h).

The study protocol was approved by the institutional review board in West China Hospital, Sichuan University, Chengdu, China. Each patient or the patient’s legal representative received oral and written information about the trial and signed an informed consent form before enrolment. The study was undertaken in full accordance with the Declaration of Helsinki and Good Clinical Practice guidelines and reported according to CONSORT (Consolidated Standards of Reporting Trials) guidelines. The study protocol is available in Supplementary Appendix. This trial was registered in Chinese Clinical Trial Registry (ChiCTR2000029638).

### Randomisation and masking

Eligible patients were randomized in a 1:1 ratio using a computer-generated random number table to the rSIFN-co group or the interferon-alpha group. The study medications were prepared by the medical ward nurses and then dispensed to the participants. Patients were blinded to treatment allocation, whereas treating physicians were aware of group allocations.

### Interventions

Patients received nebulized rSIFN-co (12 IU, twice daily) or nebulized interferon-alpha (interferon-alpha-2a or interferon-alpha-2b, 5 million IU, twice daily) immediately after randomization until discharged from the hospital, but not more than 28 days. The baseline antiviral agents were lopinavir-ritonavir (400 mg and 100 mg, orally, twice daily) or umifenovir (200 mg, orally, thrice daily), which were freely provided by the national health authority. All patients received the standard care as well as the interferon treatment, and were subjected to the laboratory, and radiographic examinations. Clinical, laboratory, and radiographic assessments were conducted at baseline. Patients were assessed once daily by trained researchers from day 0 to day 28. A complete blood count, serum biochemical tests (renal function, liver function), and a nasopharyngeal swab test for SARS-CoV-2 using the RT-PCR assay (approved by the National Medical Products Administration) were conducted every 3 days, while chest CT scans were conducted every 5 or 7 days. The virus nucleic acid negative conversion was defined as two consecutive negative tests for SARS-CoV-2 (interval of more than 24 h). Moreover, chest CT scans was graded by the changed areas of ground-glass opacity and consolidation compared with the baseline by two independent radiologists. Data were collected and recorded on paper case report forms and then entered into an electronic database and validated by trial staffs.

### Outcome measures

The primary endpoint was the time to clinical improvement, defined as the time from enrolment to an improvement of two points on a seven-category ordinal scale [19] (Supplementary Table 2) or live discharge from the hospital, whichever came first.

Secondary endpoints included the time to radiological improvement defined as the time from enrolment to radiological improvement on chest CT scans, the time to virus nucleic acid negative conversion defined as the time from enrolment to two consecutive negative tests for SARS-CoV-2 *via* RT-PCR testing on nasopharyngeal swabs samples, the overall rate of clinical improvement assessed on day 28. Safety outcomes included treatment-emergent adverse events (AEs) and severe adverse events (SAEs), classified according to the National Cancer Institute Common Terminology Criteria for Adverse Events, version 4.0. AEs and SAEs were assessed and recorded once daily by trained researchers from day 0 to day 28. Other secondary outcomes included overall rates of radiological improvement on days 7, 14, and 28 on chest CT scans, overall rates of virus nucleic acid negative conversion *via* RT-PCR testing on nasopharyngeal swabs samples on days 7, 14, and 28, and the rates of deterioration or death on day 28.

### Statistical analysis

This trial was designed as an exploratory one and was not powered statistically to measure a specific outcome, thus sample size estimates were not based on statistical power assessments. All participants who received study medications at least once were included in the safety analysis. The time to clinical improvement was assessed after all patients had reached day 28. Patients that failed to reach clinical improvement or died before day 28 were considered as right-censored. We used the Kaplan–Meier method to analyze the time to events in the safety population with a log-rank test. We used a Cox model to estimate the hazard ratios (HRs) with 95% confidence intervals (CIs). We used the rate differences or median differences between groups to compare the event rates of secondary or other outcomes. A two-sided α of less than 0.05 was considered statistically significant. We used the StataSE software, version 14.0 for statistical analysis.

## Results

**Figure 1.**
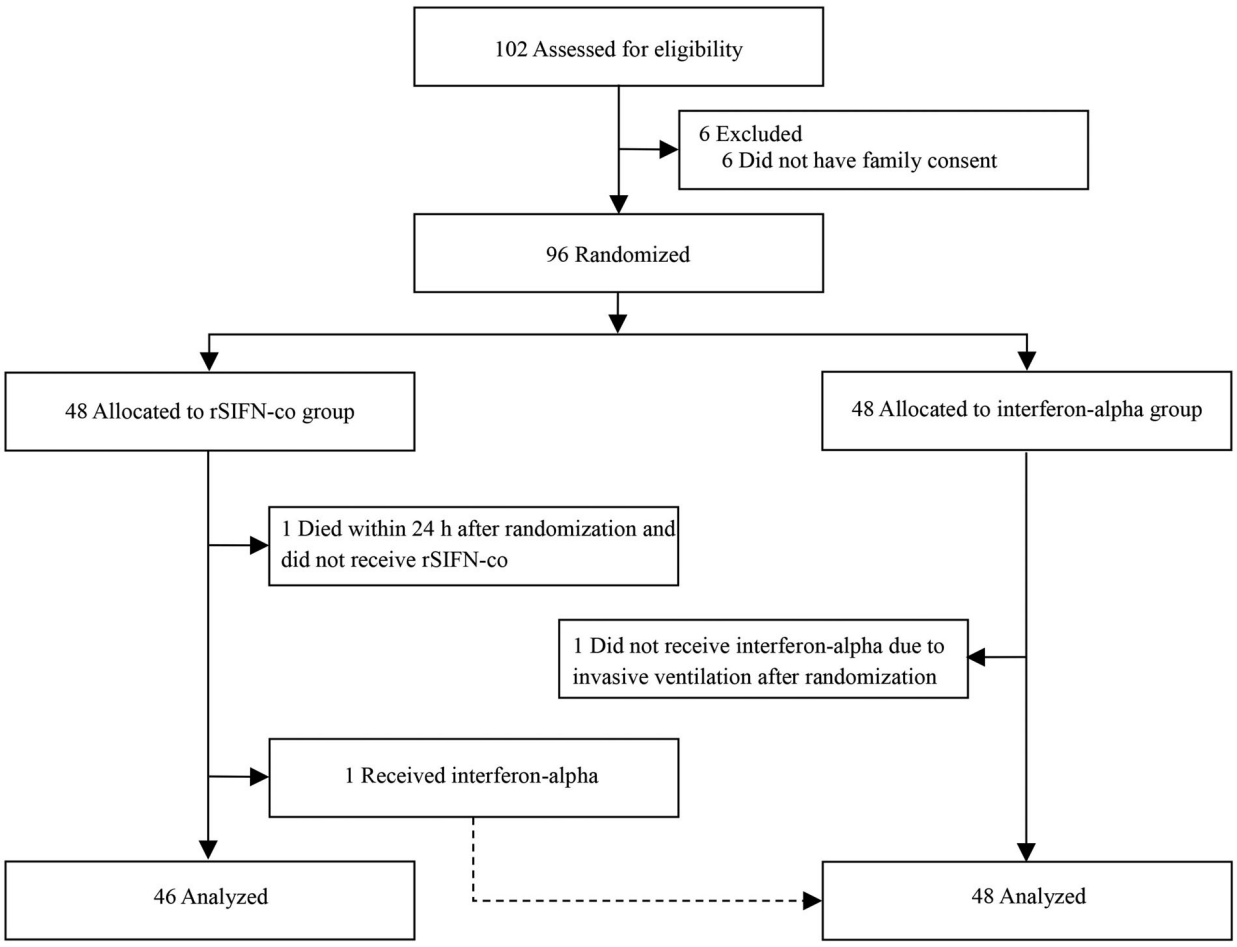
Trial profile.

A total of 102 patients with COVID-19 from five hospitals in China were recruited and assessed for eligibility. Six patients did not have family consent and the remaining 96 patients were randomly divided into two groups (48 in each group) (Figure 1). In the rSIFN-co group, two patients were excluded (one died within 24 h after randomization, and the other was prescribed interferon-alpha instead of rSIFN-co due to the attending physician’s misinterpretation of the randomization result). One patient in the interferon-alpha group did not receive interferon-alpha because of acute exacerbation of the disease and administration of invasive ventilation. Finally, 46 and 48 patients were included in the rSIFN-co group and interferon-alpha group, respectively, for evaluation. The study groups were similar at baseline in terms of demographic characteristics, laboratory test results, distribution of ordinal scale scores, chest CT results, patients’ status and therapeutics received after enrolment (Tables 1 and 2).

**Table 1.** Baseline demographic, clinical, laboratory, and radiographic characteristics of the study population.

Characteristic	Total (*n* = 94)	rSIFN-co (*n* = 46)	Interferon-alpha (*n* = 48)
Age (years)	54.0 (39.8–63.3)	51.0 (33.5–59.3)	56.0 (49.3–69.0)
Male sex	44 (46.8)	21 (45.7)	23 (47.9)
Component			
Moderate	83 (88.3)	39 (84.8)	44 (91.7)
Severe	11 (11.7)	7 (15.2)	5 (10.4)
Comorbidities			
Hypertension	18 (19.1)	7 (15.2)	11 (22.9)
Diabetes	9 (9.6)	3 (6.5)	6 (12.5)
Heart disease	7 (7.4)	3 (6.5)	4 (8.3)
Cerebrovascular disease	5 (5.3)	3 (6.5)	2 (4.2)
Tuberculosis	3 (3.2)	3 (6.5)	0
Liver diseases	6 (6.4)	3 (6.5)	3 (6.3)
COPD	1 (1.1)	1 (2.2)	0
Body temperature (°C)	36.7 (36.4–36.9)	36.8 (36.5–37.0)	36.7 (36.4–36.9)
Fever	15 (16.0)	8 (17.4)	7 (14.6)
Cough	51 (54.3)	26 (56.5)	25 (52.1)
Expectoration	21 (22.3)	10 (21.7)	11 (22.9)
Fatigue	21 (22.3)	11 (23.9)	10 (20.8)
Myalgia	12 (12.8)	7 (15.2)	5 (10.4)
Anhelation	15 (16.0)	6 (13.0)	9 (18.8)
Dyspnoea	8 (8.5)	5 (10.9)	3 (6.3)
Pharyngalgia	7 (7.4)	4 (8.7)	3 (6.3)
Poor appetite	7 (7.4)	3 (6.5)	4 (8.3)
Diarrhoea	5 (5.3)	2 (4.3)	3 (6.3)
Other	7 (7.4)	4 (8.7)	3 (6.3)
Respiratory rate (breaths per min)	20 (18.8–20.0)	20 (18.0–20.0)	20 (19.0–20.0)
Respiratory rat*e* > 24 breaths per min	6 (6.4)	3 (6.5)	3 (6.3)
Heart rate (beats per min)	82.5 (77.8–93.0)	82.0 (77.6–90.0)	83.0 (77.3–93.0)
Oxygen saturation	98.0 (97.0–99.0)	98.0 (97.0–99.0)	98.0 (96.3–98.0)
Oxygen saturatio*n* < 94%	3 (3.2)	2 (4.3)	1 (2.1)
White blood cell count (×10^−9^ /L)	5.4 (4.4–6.8)	5.4 (4.6–6.8)	5.5 (4.2–6.8)
4.0–10.0	76 (80.9)	39 (84.8)	39 (81.2)
<4.0	16 (17.0)	7 (15.2)	9 (18.8)
>10.0	2 (2.1)	0	2 (4.2)
Lymphocyte count (×10^−9^/L)	1.5 (1.1–1.8)	1.5 (1.1–1.8)	1.37 (0.9–1.8)
≥1.0	75 (79.8)	39 (84.8)	36 (75.0)
<1.0	19 (20.2)	7 (15.2)	12 (25.0)
Platelet count (10^−9^ /L)	206 (167.5–251.3)	208 (176.5–247.8)	198.5 (155.0–260.8)
≥100	89 (94.7)	44 (95.7)	45 (93.7)
<100	5 (5.3)	2 (4.3)	3 (6.3)
Serum creatinine (μmol/L)	69.0 (54.0–81.5)	65.5 (51.8–76.7)	71.9 (59.5–94.5)
≤133	90 (95.7)	46 (100.0)	44 (91.7)
>133	4 (4.3)	0	4 (8.3)
Alanine aminotransferase (U/L)	21.0 (13.9–34.8)	23.7 (17.9–50.8)	16.7 (11.4–28.3)
≤50	79 (84.0)	35 (76.1)	44 (91.7)
>50	15 (16.0)	11 (23.9)	4 (8.3)
Aspartate aminotransferase (U/L)	20.8 (15.0–28.3)	23.5 (15.5–34.4)	18.4 (13.9–24.5)
≤40	80 (85.1)	37 (80.4)	43 (89.6)
>40	14 (14.9)	9 (19.6)	5 (10.4)
Creatine kinase (U/L)	56.0 (42.8–82.7)	56.0 (40.3–73.7)	57.0 (43.7–93.0)
≤185.0	88 (93.6)	44 (95.7)	44 (91.7)
> 185.0	6 (6.4)	2 (4.3)	4 (8.3)
C–reactive protein (mg/Dl)	4.2 (1.1–4.2)	3.1 (1.0–15.8)	6.1 (1.3–29.1)
Chest CT scans			
Ground-glass opacity infiltration	66 (70.2)	34 (73.9)	32 (66.7)
Unilateral	11 (11.7)	8 (17.4)	3 (6.3)
Bilateral	55 (58.5)	26 (56.5)	29 (60.4)
Consolidation	34 (36.2)	21 (45.7)	12 (25.0)
Unilateral	4 (4.3)	2 (4.3)	2 (4.2)
Bilateral	30 (31.9)	20 (43.5)	10 (20.8)
Pleural effusion	7 (7.4)	2 (4.3)	5 (10.4)

Data are *n* (%) or median (IQR). Fever was defined as body temperature ≥ 37.3 °C.

**Table 2.** Patients’ status and treatments received after enrolment.

Characteristic	**rSIFN-co** **(*n* = 46)**	**Interferon-alpha** **(*n* = 48)**
Days from illness onset to randomisation (days)	14.0 (5.0–30.0)	14.5 (7.0–31.0)
Seven-category scale on day 1		
3: Hospitalisation, not requiring supplemental oxygen	11 (23.9)	11 (22.9)
4: Hospitalisation, requiring supplemental oxygen	20 (43.5)	28 (58.3)
5: Hospitalisation, requiring high-flow nasal cannula or non-invasive mechanical ventilation	15 (32.6)	9 (18.8)
Receiving lopinavir–ritonavir	22 (47.8)	20 (41.7)
Receiving umifenovir	24 (52.2)	28 (58.3)
Oxygen therapy support	35 (76.1)	42 (87.5)
Nasal cannula	32 (69.6)	39 (81.3)
Mask	2 (4.3)	2 (4.2)
Non-invasive mechanical ventilation	1 (2.2)	0
Invasive mechanical ventilation	0	1 (2.1)
Duration of oxygen support	12.0 (10.0–16.0)	14.0 (10.0–17.5)
Antibiotic	13 (28.3)	8 (16.7)
Glucocorticoid therapy	4 (6.5)	5 (10.4)
Duration of glucocorticoid therapy	5.5 (4.0–7.8)	4.0 (3.0–6.5)
Immunoglobulins	2 (4.3)	1 (2.1)

Data are *n* (%) or median (IQR).

The median age of patients was 54.0 years (interquartile range [IQR] 39.8 to 63.3), and 46.8% of the patients were male. In the rSIFN-co group there were 39 moderate cases and 7 severe cases, while in the interferon-alpha group, there were 43 moderate cases and 5 severe cases. The median interval between symptom onset and randomization was 14.0 days (IQR 5.0 to 30.0) in the rSIFN-co group and 14.5 days (IQR 7.0 to 31.0) in the interferon-alpha group. Some patients (rSIFN-co, *n* = 26; interferon-alpha, *n* = 27) received symptomatic treatment (such as cough relief and fever reduction) and/or supportive care before randomization. All patients received lopinavir-ritonavir or umifenovir at baseline. During the trial, nine patients received systemic glucocorticoids (four in the rSIFN-co group and five in the interferon-alpha group).

One patient in the interferon-alpha group suffered disease deterioration and was transferred to another hospital, which was designated for critical ill patients. The other 10 patients (rSIFN-co, *n* = 2; interferon-alpha, *n* = 8) were transferred to other hospitals according to the government’s unified deployment. The detailed information about the status of these patients when transferred are summarized in Supplementary Table 3. The outcomes of these 11 patients were assessed when transferred and included in the final analysis.

**Figure 2.**
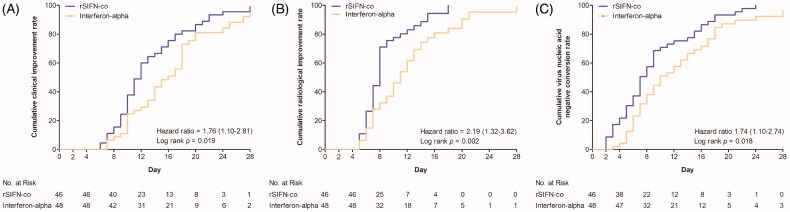
Outcomes over time. (A) Time to clinical improvement; (B) Time to radiological improvement on chest CT scans; (C) Time to virus negative conversion. Analysis was performed by log-rank (Mantel-Cox) test.

The time to clinical improvement in the rSIFN-co group was statistically shorter than that in the interferon-alpha group (median, 11.5 days vs 14.0 days; HR, 1.76; 95% CI, 1.10 to 2.81; *p* = .019) (Table 3 and Figure 2).

**Table 3.** Outcomes in the study population.

Characteristic	rSIFN-co (*n* = 46)	Interferon-alpha (*n* = 48)	Difference
Seven-category scale on day 7			
2: Not hospitalised, but unable to resume normal activities	6 (13.0)	6 (12.5)	
3: Hospitalisation, not requiring supplemental oxygen	9 (19.6)	12 (25.0)	
4: Hospitalisation, requiring supplemental oxygen	23 (50.0)	21 (43.8)	
5: Hospitalisation, requiring high-flow nasal cannula or non-invasive mechanical ventilation	8 (17.4)	8 (16.7)	
6: Hospitalisation, requiring extracorporeal membrane oxygenation, invasive mechanical ventilation, or both	0	1 (2.0)	
Seven-category scale on day 14			
2: Not hospitalised, but unable to resume normal activities	27 (58.7)	21 (43.8)	
3: Hospitalisation, not requiring supplemental oxygen	10 (21.7)	15 (31.2)	
4: Hospitalisation, requiring supplemental oxygen	8 (17.4)	8 (16.7)	
5: Hospitalisation, requiring high-flow nasal cannula or non-invasive mechanical ventilation	1 (2.2)	3 (6.3)	
6: Hospitalisation, requiring extracorporeal membrane oxygenation, invasive mechanical ventilation, or both	0	1 (2.0)	
Seven-category scale on day 28			
2: Not hospitalised, but unable to resume normal activities	44 (95.6)	44 (91.6)	
3: Hospitalisation, not requiring supplemental oxygen	1 (2.2)	1 (2.1)	
4: Hospitalisation, requiring supplemental oxygen	1 (2.2)	2 (4.2)	
5: Hospitalisation, requiring high-flow nasal cannula or non-invasive mechanical ventilation	0	0	
6: Hospitalisation, requiring extracorporeal membrane oxygenation, invasive mechanical ventilation, or both	0	1 (2.1)	
Time to clinical improvement (days)	11.5 (9.3–16.0)	14.0 (10.0–18.0)	1.76 (1.10–2.81)
Clinical improvement rates			
Day 7	5 (10.9)	3 (6.3)	4.6 (−0.07–0.16)
Day 14	30 (65.2)	19 (39.6)	25.6 (0.06–0.45)
Day 28	43 (93.5)	37 (77.1)	16.4 (0.03–0.30)
Time to radiological improvement (days)	8.0 (6.0–8.3)	10.0 (7.0–13.0)	2.19 (1.32–3.62)
Radiological improvement rates			
Day 7	20 (43.5)	13 (25.0)	18.5 (−0.03–0.35)
Day 14	39 (84.8)	32 (66.7)	18.1 (0.01–0.35)
Day 28	42 (91.3)	38 (79.2)	12.1 (−0.02–0.26)
Time to virus nucleic acid negative conversion (days)	7.0 (5.0–13.0)	10.0 (6.3–16.8)	1.74 (1.10–2.74)
Virus nucleic acid negative conversion rates			
Day 7	23 (50.0)	15 (31.3)	18.7 (−0.01–0.38)
Day 14	35 (76.1)	31 (64.6)	11.5 (−0.07–0.30)
Day 28	45 (97.8)	41 (85.4)	12.4 (0.02–0.23)
Day 28 mortality	0	0	−
Deterioration rates	0	1 (2.1)	−2.1 (−0.08–0.04)

Data are *n* (%) or median (IQR). The hazard ratio was estimated by Cox model for time to events. Differences were expressed as rate differences and 95% confidence intervals for the overall rates of clinical improvement, radiological improvement on chest CT scans and virus nucleic acid negative conversion on days 7, 14, and 28 and deterioration rate.

The overall rate of clinical improvement on day 28 was much higher in the rSIFN-co group than that in the interferon-alpha group (93.5% vs 77.1%; difference, 16.4 percentage points; 95% CI, 3% to 30%) (Table 3). The time to radiological improvement in the rSIFN-co group was significantly shorter than that in the interferon-alpha group (median, 8.0 days vs 10.0 days; HR, 2.19; 95% CI, 1.32 to 3.62; *p* = .002) (Table 3 and Figure 2). The time to virus nucleic acid negative conversion in the rSIFN-co group was also significantly shorter than that in the interferon-alpha group (median 7.0 days vs 10.0 days; HR, 1.74; 95% CI 1.10 to 2.74; *p* = .018) (Table 3 and Figure 2). The overall rates of radiological improvement on chest CT scans on days 7 and 28 were numerically higher in the rSIFN-co group than those in the interferon-alpha group, however a significant difference was only observed between the two groups on day 14 (84.8% vs 66.7%; difference, 18.1 percentage points; 95% CI, 1% to 35%) (Table 3). The overall rate of virus nucleic acid negative conversion on day 28 was much higher in the rSIFN-co group than that in the interferon-alpha group (97.8% vs 85.4%; difference, 12.4 percentage points; 95% CI, 2% to 23%), while the overall rates on day 7 and 14 in the rSIFN-co group were numerically higher than those in the interferon-alpha group (Table 3). One patient in the interferon-alpha group experienced a secondary bacterial infection and developed respiratory failure on day 6. Tracheal intubation and mechanical ventilation were applied to her. She was transferred to another hospital for further treatment on day 10.

AEs were reported by 13 (28.3%) of the 46 patients in the rSIFN-co group and 18 (38.5%) of the 48 patients in the interferon-alpha group (Supplementary Table 4), most of which were classified as grade 1 or 2 with decreased appetite being the most common in both groups. There were no SAEs reported in the rSIFN-co group, while one patient had a secondary bacterial infection followed by respiratory failure in the interferon-alpha group. The latter case was deemed to be a non-treatment related SAE, and interferon-alpha administration was ceased and invasive mechanical ventilation was applied to this patient. There were no deaths in either group between the initiation of medication and day 28.

## Discussion

In this multicenter, head-to-head, randomized controlled trial, the combination of rSIFN-co nebulization and antiviral agents significantly improved the recovery in moderate-to-severe patients with COVID-19 as compared with the combination of interferon-alpha and antiviral agents. This benefit was seen in shortening the time to clinical improvement, time to radiological improvement, and time to virus nucleic acid negative conversion. Additionally, the clinical improvement rate on day 28 was also significantly higher in the rSIFN-co group than that in the interferon-alpha group.

This trial did not enrol any mild or critically ill patients because this was an exploratory study. Instead, we recruited moderate-to-severe patients with COVID-19. Our population mainly consisted of moderate COVID-19 (88.3%) and no patient was given invasive mechanical ventilation or extracorporeal membrane oxygenation at the time of enrolment. During the study period, patients with COVID-19 were admitted to hospitals of different levels according to the severity of the disease following the guidance of health administration department. Our patients were recruited from five hospitals designated for moderate-to-severe patients. The time interval between symptom onset and randomization varied among patients, and some of the participants had received symptomatic treatment and/or supportive care, but without clinical improvement. We distributed the participants evenly between the two groups by randomization. Although some patients were transferred during the middle of the study and failed to complete the whole treatment regimen according to the government’s unified deployment, all of them were evaluated before transfer and were included in the final analysis.

Interferon-alphas alone or combined with other antiviral agents have antiviral effects on multiple types of viral infections, such as SARS-CoV and MERS-CoV [11,12,20–23]. Findings from a preliminary, uncontrolled study revealed that interferon-alphacon-1 plus corticosteroids was associated with reduced disease-associated impaired oxygen saturation, more rapid resolution of radiographic lung abnormalities in SARS patients, as well as interferon beta combined with ribavirin demonstrating antiviral activity against MERS [11,12]. In addition, SARS-CoV-2 is homologous with MERS-CoV and SARS-CoV and presenting similar properties, combination antiviral therapy with interferon-alpha may be effective for COVID-19 [24,25]. rSIFN-co is a new homolog of interferon-alpha and not yet commercialized, it has shown stronger antiviral effects and less side effects during preclinical use compared with traditional interferons [14–18]. Thus, we inferred that rSIFN-co might be a potential therapeutics and more efficient than traditional interferon-alpha in the treatment of COVID-19, and conducted this exploratory, head-to-head trial. Although there were no antiviral agents confirmed to be effective during the study period, all participants in this study still received baseline antiviral agents (lopinavir–ritonavir or umifenovir) to make sure that all of the patients could benefit from any potential therapeutics.

However, severe COVID-19 patients often develop acute respiratory distress syndrome (ARDS) or secondary haemophagocytic lymphohistiocytosis (sHLH) [26,27]. Both ARDS and sHLH are hallmarks of overwhelmed cytokine productions, so called cytokine storm or cytokine release syndrome (CRS), which is one of main causes of mortality [28,29]. Therefore, it has been controversial in clinic whether interferon-alpha alone should be used for treating high pathological viruses, such as SARS-CoV-2, SARS-CoV and MERS-CoV, although interferon-alpha were empirically recommended as one of therapeutic option for COVID-19 in clinical practice [13]. In both animal studies and clinic, the early treatment of interferon rescued mice from lethal doses of SARS-CoV and MERS and early administration of interferon-alpha 2 might be promising for COVID-19 patients, especially in those who demonstrate a defective interferon response, however, late interferon administration delayed viral clearance and exacerbate immunopathology [30–33]. In supporting the notion of anti-cytokine storm may be beneficial to COVID-19 patients, the administration of anti-inflammation drug, methylprednisolone, slowed down the disease progress and reduced dealth rate [34]. On the other hand, our study suggest that treatment of moderate-to-severe COVID-19 patients with interferon can ameliorate clinical outcomes. This result may due to the nature of SARS-CoV-2 and related virus infections, such as SARS and MERS, to dysregulation of interferon-alpha induction at early stage of infection [35].

Furthermore, studies showed type I interferon deficiency could be a hallmark of COVID-19 and at least 10% of patients with COVID-19 pneumonia have neutralizing auto-antibodies against type I interferons, which highlights the crucial role of type I interferons in protective immunity against SARS-CoV-2 [36,37]. A randomized controlled trial (RCT) first confirmed that the combination of interferon beta-1b and antiviral agents accelerated the recovery of patients with mild-to-moderate COVID-19 compared with single antiviral agent alone. They suggested that interferon-beta-1b appeared to be a key component of the combination treatment in subgroup analysis [38]. A retrospective cohort study showed that using early interferon-alpha-2b could reduce in-hospital mortality and early initiation of interferons with lopinavir–ritonavir is associated with more favourable clinical responses than by using lopinavir–ritonavir alone in COVID-19 patients [39]. Another exploratory study demonstrated that interferon-alpha-2b therapy appears to shorten duration of viral shedding and reduce inflammatory markers interleukin-6 and C-reactive protein, which support the plausibility of interferon-alpha-2b representing a therapy for COVID-19 [40]. Our study is the first RCT which demonstrated that the combination of rSIFN-co and antiviral agents could reach encouraging results, even in moderate-to-severe cases. Meanwhile, our study confirmed the superiority of rSIFN-co versus interferon-alpha when used in combination with baseline antiviral agents. The overall rates of clinical improvement were 93.5% and 77.1% on day 28 in the rSIFN-co group and interferon-alpha group, respectively. Based on the fact that the baseline antiviral agents (lopinavir–ritonavir or umifenovir) may be ineffective in treating COVID-19 when used alone [8,9], we argue that the antiviral effects were mainly attributed to the interferon-alpha or synergies from the combination. These findings revealed that the combination of interferons with antiviral agents was a potential therapeutic approach for COVID-19. rSIFN-co plus lopinavir–ritonavir or umifenovir might be an effective therapeutics for treating COVID-19. Most recently, remdesivir was proven to be superior to placebo in shortening the time to recovery in adults hospitalized with COVID-19 [5]. Combination of rSIFN-co and remdesivir should be strongly expected in the future.

Previous studies on interferon-alpha showed that a few patients had influenza-like symptoms, such as pyrexia, myalgia, and rigours, after receiving treatment [20–23]. The present exploratory study demonstrated the superiority of rSIFN-co over interferon-alpha for COVID-19 patients with a low rate of AEs. No patient had influenza-like symptoms in these two groups. However, gastrointestinal AEs, including decreased appetite, nausea, diarrhoea, abdominal discomfort and stomach ache, were relatively common in this study. The incidence of gastrointestinal AEs was similar to previous studies focussing on lopinavir–ritonavir or umifenovir [8,9]. As all of the enrolled patients in this study had received treatment with the antiviral agents, the recorded AEs might be related to those compounds. In addition, one patient’s condition in the interferon-alpha group deteriorated by what was thought to be the natural progression of SARS-CoV-2 infection, this is a fairly common event in COVID-19 patients. Given these recorded AEs, we conclude that the adding either rSIFN-co or interferon-alpha nebulization as a therapeutic option to the current antiviral agents is safe. It should be noted that although rSIFN-co is a homolog of interferon-alpha, it can be used at higher doses with a low rate of AEs. This is one of the reasons that we used a high dose of rSIFN-co in this study as compared to interferon-alpha (12 million IU vs 5 million IU). The high doses of rSIFN-co might have contributed to the better outcomes observed in our study.

Our study has several limitations. Firstly, the total number of trial patients was small, although it is not uncommon for an exploratory study, further studies are encouraged to confirm these results with more patients. Secondly, the median interval between symptom onset and randomization was longer in our study than that in other reports and some patients received symptomatic treatment (such as cough relief and fever reduction) and/or supportive care before randomization [2,3]. Thirdly, we were unable to mask research staff to the treatment allocation, which might introduce potential performance bias when they were doing ordinal scale measurements. However, this was mitigated because they were trained. In addition, the secondary endpoints included some objective parameters like the time to virus nucleic acid negative conversion which also supported the clinical findings. Fourthly, dissimilar baseline concurrent therapeutics, such as antiviral agents, antibiotics, corticosteroids or immunoglobulins, might be other possible confounders, but we endeavoured to minimize these effects by randomization.

In conclusion, rSIFN-co was associated with a shorter time of clinical improvement than traditional interferon-alpha in the treatment of moderate-to-severe COVID-19 when combined with baseline antiviral agents. Larger studies with rSIFN-co therapy alone or combined with other antiviral therapy that include a broader range of patients with COVID-19 are worth to be conducted.

## Supplementary Material

Supplemental Material

## Data Availability

The authors of included studies should be contacted individually regarding requests to share individual patient data.
