# Supplementary material for: Effect of a genetically engineered interferon-alpha versus traditional interferon-alpha in the treatment of moderate-to-severe COVID-19: a randomised clinical trial
Source: Ann Med. 2021 Feb 23;53(1):391–401. doi: 10.1080/07853890.2021.1890329 (PMC7906612; doi:10.1080/07853890.2021.1890329)
Supplement: Supplemental Material [file IANN_A_1890329_SM8666.doc]

# Table S1. Clinical Classification for COVID-19 patients

|  | **Diagnostic criteria^a^** |
| --- | --- |
| Mild | The clinical symptoms were mild, and there was no sign of pneumonia on imaging. |
| Moderate | Showing fever and respiratory symptoms with radiological findings of pneumonia. |
| Severe | Adult cases meeting any of the following criteria:  (1) Respiratory distress (≥30 breaths/min);  (2) Oxygen saturation ≤93% at rest;  (3) Arterial partial pressure of oxygen (PaO2)/fraction of inspired oxygen (FiO2) ≤300 mmHg (1 mmHg = 0.133 kPa).  In high-altitude areas (at an altitude of over 1000 meters above the sea level), PaO2/FiO2 shall be corrected by the following formula:  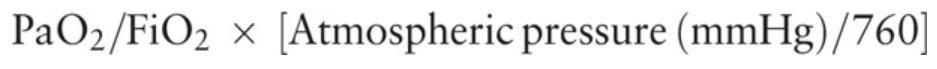  Child cases meeting any of the following criteria:   1. Tachypnea (RR ≥ 60 breaths/min for infants aged below 2 months; RR ≥ 50 BPM for infants aged 2–12 months; RR ≥ 40 BPM for children aged 1–5 years, and RR ≥ 30 BPM for children above 5 years old) independent of fever and crying; 2. Oxygen saturation ≤92% on finger pulse oximeter taken at rest; 3. Labored breathing (moaning, nasal fluttering, and infrasternal, supraclavicular, and intercostal retraction), cyanosis, and intermittent apnea; 4. Lethargy and convulsion; 5. Difficulty feeding and signs of dehydration. |
| Critical cases | Cases meeting any of the following criteria:   1. Respiratory failure and requiring mechanical ventilation; 2. Shock; 3. With other organ failure that requires Intensive Care Unit care. |
| ^a^Clinical Classification for COVID-19 patients was based on the *Diagnosis and Treatment Protocol for Novel Coronavirus Pneumonia* released by National Health Commission of China. | |

# Table S2. Seven-category ordinal scale for clinical improvement^a^

| **Point** | **Clinical Status** |
| --- | --- |
| 1 | Not hospitalized with resumption of normal activities |
| 2 | Not hospitalized, but unable to resume normal activities |
| 3 | Hospitalized, not requiring supplemental oxygen |
| 4 | Hospitalized, requiring supplemental oxygen |
| 5 | Hospitalized, requiring high-flow nasal cannula, noninvasive mechanical ventilation, or both |
| 6 | Hospitalized, requiring extracorporeal membrane oxygenation, invasive mechanical ventilation, or both |
| 7 | Death. |
| ^a^The time to clinical improvement was defined as the time from enrollment to an improvement of two points on a seven-category ordinal scale or live discharge from the hospital, whichever came first. | |

# Table S3. Characteristics of transferred patients

|  | **rSIFN-co**  **(*n* = 2)** | **Interferon alfa**  **( *n* = 9)** |
| --- | --- | --- |
| Age (years) | 75 | 69.0 (63.5-76.5) |
| Male | 1 (50.0) | 3 (33.3) |
| Duration of hospital stay before transferred (days) | 15 | 10.0 (9.5-21.5) |
| Reasons for transfer |  |  |
| Government’s unified deployment^a^ | 2 (100.0) | 8 (88.9) |
| Deterioration | 0 | 1 (11.1) |
| Seven-category scale on day 1 |  |  |
| 3: Hospitalization, not requiring supplemental oxygen | 0 | 2 (22.2) |
| 4: Hospitalization, requiring supplemental oxygen | 1 (50.0) | 5 (55.6) |
| 5: Hospitalization, requiring high-flow nasal cannula or noninvasive mechanical ventilation | 1 (50.0) | 2 (22.2) |
| Seven-category scale when transferred |  |  |
| 2: Not hospitalized, but unable to resume normal activities | 0 | 0 |
| 3: Hospitalization, not requiring supplemental oxygen | 2 (100.0) | 8 (88.9) |
| 4: Hospitalization, requiring supplemental oxygen | 0 | 0 |
| 5: Hospitalization, requiring high-flow nasal cannula or noninvasive mechanical ventilation | 0 | 0 |
| 6: Hospitalization, requiring extracorporeal membrane oxygenation, invasive mechanical ventilation, or both | 0 | 1 (11.1) |
| Chest CT scans |  |  |
| Improve | 1 (50.0) | 2 (22.2) |
| Stable | 1 (50.0) | 6 (66.7) |
| Progressive | 0 | 1 (11.1) |
| Virus nucleic acid |  |  |
| Negative | 1 (50.0) | 6 (66.7) |
| Positive | 1 (50.0) | 3 (33.3) |
| Data are n (%) or median (IQR). ^a^Government’s unified deployment: During the COVID-19 pandemic, patients with comorbidities needed to be transferred to designated hospitals even if virus nucleic acid negative conversion. Abbreviations: no.: number; IQR: interquartile range. | | |

# Table S4. Adverse events in the safety population

|  | **rSIFN-co ( *n* = 46)** |  | **Interferon alfa (*n* = 48)** | |
| --- | --- | --- | --- | --- |
|  | **Any grade** | **Grade 3 or 4** | **Any grade** | **Grade 3 or 4** |
| **Adverse events (in ≥1 patients in any treatment group)** | | | | |
| **Any adverse event** |  |  |  |  |
| Decreased appetite | 7 (15.2) | 0 | 8 (16.7) | 0 |
| Abdominal discomfort | 1 (2.2) | 0 | 6 (12.5) | 0 |
| Nausea | 3 (6.5) | 0 | 4 (8.3) | 0 |
| Lymphopenia | 2 (4.3) | 0 | 4 (8.3) | 0 |
| Diarrhea | 2 (4.3) | 0 | 3 (5.3) | 0 |
| Anemia | 3 (6.5) | 0 | 2 (4.2) | 0 |
| Hypoalbuminemia | 2 (4.3) | 0 | 1 (2.1) | 0 |
| Increased aspartate aminotransferase | 2 (4.3) | 0 | 2 (4.2) | 0 |
| Increased alanine aminotransferase | 2 (4.3) | 0 | 4 (8.3) | 0 |
| Thrombocytopenia | 2 (4.3) | 0 | 1 (2.1) | 0 |
| Increased total bilirubin | 2 (4.3) | 0 | 2 (4.2) | 0 |
| Increased cholesterol | 2 (4.3) | 0 | 1 (2.1) | 0 |
| Neutropenia | 1 (2.2) | 0 | 1 (2.1) | 0 |
| Leukopenia | 1 (2.2) | 0 | 2 (4.2) | 0 |
| Reduced serum sodium | 2 (4.3) | 0 | 1 (2.1) | 0 |
| Increased creatine kinase | 1 (2.2) | 0 | 0 | 0 |
| Sleep disorders and disturbances | 1 (2.2) | 0 | 1 (2.1) | 0 |
| Stomach ache | 0 | 0 | 1 (2.1) | 0 |
| Pharyngitis | 0 | 0 | 1 (2.1) | 0 |
| **Serious adverse event** |  |  |  |  |
| Any |  |  |  |  |
| Respiratory failure or acute respiratory  distress syndrome | 0 | 0 | 1 (2.1) | 1 (2.1) |
| Data are n (%) or median (IQR). Data include all events reported in at least one patient after randomization through day 28 after antiviral treatment. Some patients had more than one adverse event. Abbreviations: no.: number; IQR: interquartile range. | | | | |
